# Tunicate-associated bacteria show a great potential for the discovery of antimicrobial compounds

**DOI:** 10.1371/journal.pone.0213797

**Published:** 2019-03-15

**Authors:** Diah Ayuningrum, Yang Liu, Riyanti, Mada T. Sibero, Rhesi Kristiana, Meezan A. Asagabaldan, Zerlina G. Wuisan, Agus Trianto, Ocky Karna Radjasa, Agus Sabdono, Till F. Schäberle

**Affiliations:** 1 Department of Coastal Resource Management, Faculty of Fisheries and Marine Science, Diponegoro University, Semarang, Indonesia; 2 Tropical Marine Biotechnology Laboratory, Diponegoro University, Semarang, Indonesia; 3 Institute for Insect Biotechnology, Justus-Liebig-University of Giessen, Giessen, Germany; 4 Department of Bioresources of the Fraunhofer Institute for Molecular Biology and Applied Ecology, Giessen, Germany; 5 Department of Marine Science, Diponegoro University, Semarang, Indonesia; 6 Directorate of Research and Community Services, Ministry of Research, Technology and Higher Education, Jakarta, Indonesia; 7 German Center for Infection Research (DZIF), Partner Site Giessen-Marburg-Langen, Giessen, Germany; Tallinn University of Technology, ESTONIA

## Abstract

Tunicates (Ascidians, sea squirts) are marine protochordates, which live sedentary or sessile in colonial or solitary forms. These invertebrates have to protect themselves against predators and invaders. A most successful strategy, to not being eaten by predators and prevent pathogenic microorganisms to settle, is the usage of chemical molecules for defence. To accomplish this, tunicates take advantage of the specialized metabolites produced by the bacteria associated with them. Therefore, the microbiome of the tunicates can be regarded as a promising bioresource for bacterial strains producing compounds with antibacterial activity. The aim of this study was to test this hypothesis by (i) isolation of tunicate-associated bacteria, (ii) analysis of the antibacterial activities of these strains, and (iii) purification and structure elucidation of an active compound derived from this bioresource. In total, 435 bacterial strains were isolated and thereof 71 (16%) showed antibacterial activity against multidrug resistant (MDR) bacteria. Therefrom, the ethyl acetate crude extracts from liquid fermentations of 25 strains showed activity against MDR Extended-Spectrum Beta-Lactamase (MDR-ESBL) *Escherichia coli*, MDR *Bacillus cereus*, *Micrococcus luteus*, and *Bacillus megaterium*. Phenotypic analysis based on 16S rDNA sequencing revealed the active strains belonging to different genera and phyla, like *Bacillus*, *Pantoea*, *Pseudoalteromonas*, *Salinicola*, *Streptomyces*, *Vibrio* and *Virgibacillus*. To obtain first insights into the molecules responsible for the antibacterial activities observed, strain *Pseudoalteromonas rubra* TKJD 22 was selected for large-scale fermentation and the active compound was isolated. This allowed the purification and structure elucidation of isatin, a compound known for its strong biological effects, thereunder inhibition of Gram-positive and Gram-negative pathogens.

## Introduction

The exploration of new bioresources for the discovery of novel antibiotic lead structures is of importance due to the increase of antibiotic resistant pathogenic bacteria [1, 2]. In nowadays, already a multitude of pathogenic bacteria have become resistant to many kinds of antibiotics, summarized under the term Multi-Drug Resistant (MDR) bacteria [3, 4]. Cases of infection are increasing each year, leading to serious problems in clinical as well as economic aspects [5–8]. The antibiotics available for treatment of bacterial infections in hospital and outpatient settings are no more effective to combat MDR strains [9, 10]. Therefore, novel compounds must be developed and exploration of new resources can be regarded as a must.

The marine environment is the largest habitat on earth, representing more than 70% of the planet´s surface. However, it still must be regarded as understudied and unexplored if compared with terrestrial habitats [11]. Indonesia, having the second largest marine environment, is a major house of diverse and unique marine invertebrates; thereby, promoting the potential for diverse chemical substances with putative bio(techno)logical activities and applications [12–14]. The Marine National Park of Karimunjawa is a conservation area with good water conditions and an intact marine biota [15]. The latter should be regarded as a main target for the discovery of novel marine natural products, since their associated microbiomes occupy a unique niche in the ocean´s biota.

One well-investigated subphylum of marine protochordata are tunicates. Already in the early 70ies, even before Marine Natural Products have been named as a scientific discipline [16], Sigel and colleagues reported that the ethanolic extract from the Caribbean tunicate *Ecteinascidia turbinata* possessed anti-proliferative properties [17]. Later, exactly from this tunicate species, an anti-proliferative compound was isolated, which was the lead to develop the anticancer drug Yondelis (Trabectedin/ ET-743) [18]. The latter molecule shows structural similarities with the natural products saframycin and safracin, which were isolated from *Pseudomonas fluorescens*. This fact pointed towards the fact that bacteria associated with the macroorganism represent the original producer [19]. This hypothesis was finally verified by the discovery of a biosynthetic gene cluster, encoding the biosynthetic machinery responsible for ET-743 biosynthesis in the bacterial symbiont *Candidatus Endoecteinascidia frumentesis*, a γ-proteobacterium [20]. The fact that microbes associated with invertebrates are the producers of molecules of interest became well known by further examples and is also advantageous for bioprospecting approaches, since they will be more sustainable and cultivation will be possible, rendering the process faster, easier and less expensive [21].

## Materials and methods

### Sample collection and preliminary identification of the tunicates

**Fig 1:**
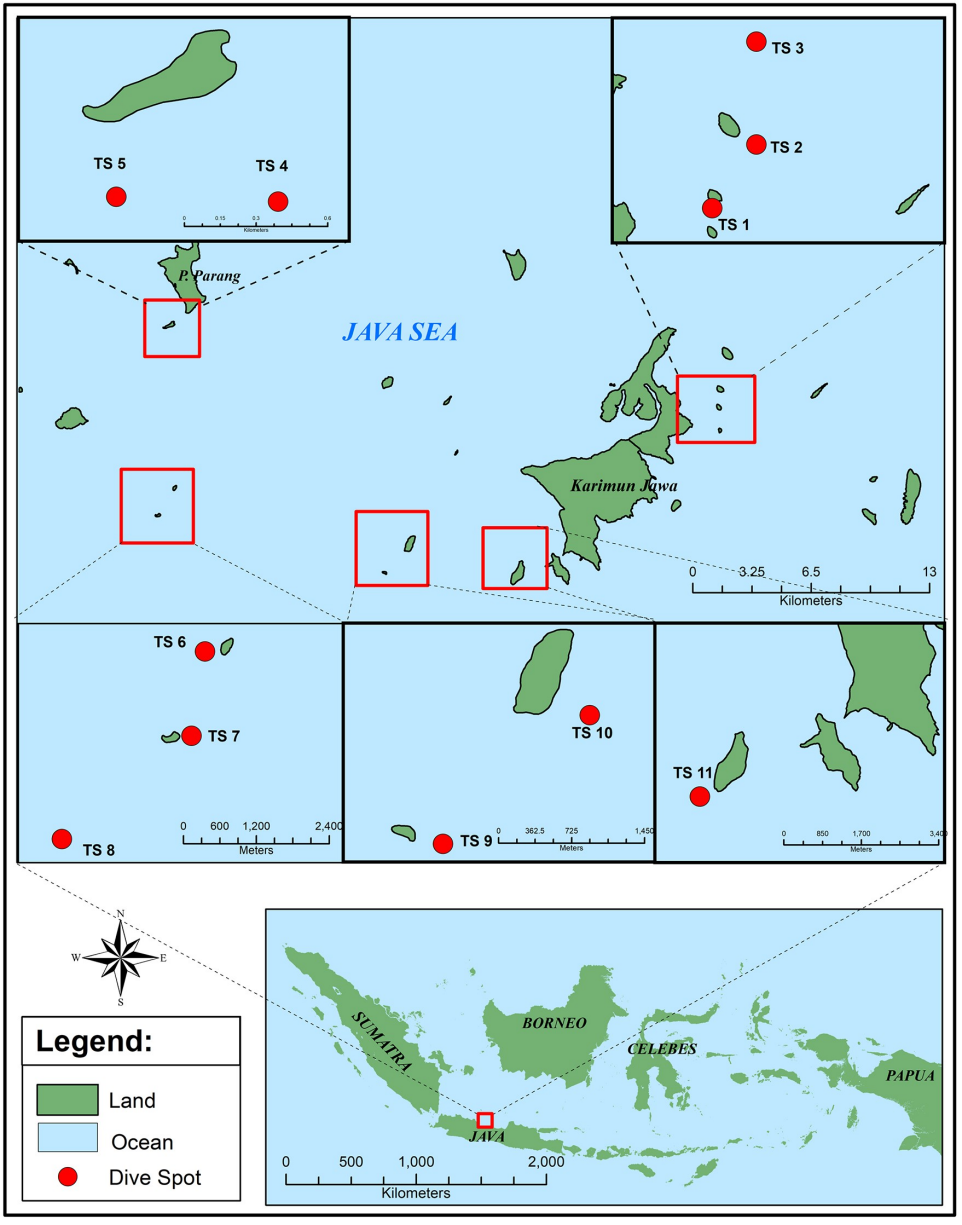
Sampling site in Karimunjawa Sea, Indonesia. TS1-5, and TS11 represent diving sites at sampling site 1 (March 2017). TS 6,7,8 represent diving sites at sampling site 2 (May 2017) and TS 9,10 represent the last sampling site (July 2017).

Sampling was performed at the Marine National Park of Karimunjawa, North Java Sea, Indonesia in March, May and July 2017 (Fig 1). All samples were collected by scuba diving at 10–18 m depths. Underwater photo documentation and labelling was conducted before the samples were stored in sterile plastic zip lock bags and cooled. They were either directly processed, or cooled until further processing in the laboratory. Preliminary identification of the specimen was based on the morphological appearance of the specimen, according to the tunicate atlas [22, 23].

### Isolation of associated bacteria

First, the environmental samples were processed under sterile condition as follows: 1 g of tunicate sample tissue was rinsed 3 times with sterile natural seawater, grinded with a sterile mortar and homogenized with 5 mL sterile natural seawater. The homogenized tunicate tissue was serially diluted and spread (30 μL) onto different media (PYA, ISP2, M1). The composition of the media used were (i) PYA medium: peptone (2.5 g), yeast extract (0.5 g), and agar (15 g), ingredients were mixed with sterile natural seawater (1 L); (ii) ISP2 medium: malt extract (10 g), yeast extract (4 g), glucose (4 g) and agar (15 g), ingredients were mixed with sterile natural sea water (1 L); (iii) M1 medium: starch (10 g), yeast extract (4 g), peptone (2 g), agar (15 g), mixed with sterile natural seawater (1 L). All plates were incubated for 2 days for fast growing bacteria and 7 days for slow growing bacteria (e.g., actinobacteria) at room temperature (24±2 °C). According to the morphological features, colonies were randomly picked to agar plates containing the same medium as before and purified using the streak plate method [24]. Purification on agar plates was continued until axenic cultures were obtained. The resulting bacterial strains were provisionally named with the initial according to the tunicate they were isolated from.

### Screening for antibacterial activities

To investigate the potential of the isolated bacterial strains for the production of compounds inhibiting the growth of MDR pathogenic bacteria, the antibacterial activity of the strains was tested against clinical isolates, which were obtained from the Medical Microbiology Laboratory Dr. Kariadi Hospital, Semarang, Central Java, Indonesia. Therefore, the isolates were incubated for 6 days on peptone yeast agar with natural seawater. Agar blocks (diameter: 6 mm) were taken from these grown cultures and were placed on Mueller Hinton Agar (MHA), which was inoculated with 30 μL of a solution containing 10^8^ CFU of the pathogen to be tested. The human pathogens used were ESBL-MDR *Escherichia coli*, MDR *Bacillus cereus*, and MDR *Escherichia coli*. Cultures were incubated at 37 °C for 24 h, after an initial incubation of 1 h at 4 °C. Antibacterial potential was determined by measuring the complete zone of inhibition (ZOI, in mm) [25]. A ZOI of 7–11 mm was categorized as low, a ZOI of 12–16 mm as moderate, a ZOI of 17–21 mm as strong, and a ZOI ≥ 21 mm as very strong antimicrobial activity.

### Generation of extracts

Bacteria were inoculated from solid medium in 20 mL Erlenmeyer flasks containing 5 mL of Marine Broth Difco medium, and fermentation was done for 24 h at 30 °C. From this pre-culture 1% (v/v) was transferred to 100 mL of the same medium in 300 mL Erlenmeyer flasks. Incubation was carried out at 30 °C for 2 days, shaking at 110 rpm. Afterwards, 100 mL ethyl acetate were added to the culture broth and the mixture was shaken thoroughly. The organic phase was collected using a separation funnel, transferred to a round bottom flask and evaporated until complete dryness under reduced pressure. The resulting extracts were dissolved to 10 mg/mL in ethyl acetate for antibacterial assays and to 1 mg/mL in methanol for LC-HRMS analysis.

### Antibacterial assays

Agar diffusion assay: LB agar plates (10g peptone, 5g yeast, 5g NaCl, 15 g agar, mixed with 1 L distilled water) were prepared and the respective test bacteria were swapped on to it. 15 μl of an ethyl acetate extract (10 mg/mL) were added to a paper disk, which was positioned on the agar plate. Incubation was performed at 37 °C overnight.

Microtiter plate assay: Samples were adjusted to a concentration of 5 mg/mL in HPLC grade MeOH and 100 μL were injected. Separation was achieved using a EC 250/4.6 Nucleodur C18 Gravity-SB 5μ REF 760619.46 column with a H_2_O:MeOH gradient (0–5 min 5% MeOH, 5–30 min gradient from 5 to 100% MeOH, 30–45 min 100% MeOH; flow rate of 0.8 mL/min). Every 30 s, equivalent to 400 μL, were collected in one well, and the microtiter plates were dried completely. Then, medium inoculated with the respective test bacteria (OD_600_ adjusted to 0.1, 200 μL per well) was added. Incubation was performed shaking at 220 rpm and 30 °C, and for the read out of the assay, a monochromator CLARIOstar BMG LABTECH’s microplate reader was used. Antimicrobial activity was determined by comparison of the OD_600_ before and after overnight incubation. The growth rate of the negative control (8 μL of the solvent ethyl acetate) was set to 100%. A final OD_600_ value <50% was considered as positive result. Carbenicillin was used as positive control.

### Identification of the bacteria and phylogenetic analysis

The bacteria were identified by 16S r DNA gene sequencing. The amplificate was either generated directly by colony PCR, or by using isolated genomic DNA as template. For the latter case, DNA extraction was performed using the DNA extraction kit from Analytik Jena following the manufacturer´s protocol. The PCR reaction (total volume 40 μL) contained 1 μL dNTPs, 2 μL of each primer, 4 μL of 10x Dream Taq Buffer (includes 20 mM MgCl_2_), 0.2 μL of GoTaq G2 Flexi DNA Polymerase (Promega, Madison, USA), 26.8 μL ddH_2_O, 2 μL DMSO and 2 μL DNA template. Primers used: PA 5’-AGA GTT TGA TCC TGG CTC AG-3’ and PH 5’-AAG GAG GTG ATC CAG CCG CA-3’, or if the first pair did not provide good results: 27F 5’-AGAGTTTGATCMTGGCTCAG-3 and 1492R 5’-GGTTACCTTGTTACGACTT-3’ [26]. PCR was done in a Biometra TRIO Thermal Cycler (Analytik Jena) using the following program: initial denaturation at 95 °C for 10 min; 34 cycles of denaturation at 95 °C for 45 s, annealing at 50 °C for 60 s, extension at 72 °C for 90 s; final extension at 72 °C for 5 min. PCR results were examined on a 1% agarose gel, visualized and documented using an INTAS UVIDoc instrument. The amplificate with the desired size of ~1.5 kbp was excised from the gel and purification was done using the Promega SV Gel and PCR Clean-Up System kit. Purified amplificates were sent to Eurofins Genomics for sequencing. The program Bioedit was used to assemble the two sequences before BLAST search, which resulted in determination of the closest relative strain, based on 16S rDNA comparison. The phylogenetic tree of the bacterial strains was constructed by the Neighbor-joining analysis, using the program MEGA 6.0 and bootstrap values are given.

### Medium optimization and large-scale cultivation

To optimize production of the antibacterial compounds, an OSMAC approach using the media MB, ISP2, SNB, NB, TSB, M1, MYE, PYB was performed. The composition of media was as follows. (i) MB (Marine Broth Carl Roth GmbH+Co.KG, Karlsruhe, Germany): 40 g mixed with distilled water 1 L, (ii) ISP2: malt extract (10 g), yeast extract (4 g), glucose (4 g) and artificial sea water (ASW) 1 L; (iii) Medium SNB (Starch Nitrate Broth): starch (20 g), KNO_3_ (1 g), K_2_HPO_4_ (0.5 g), MgSO_4_·7H_2_O (0.5 g), NaCl (0.5 g), FeSO_4_·7H_2_O (0.01 g) mixed with 1 L ASW; (iv) Medium NB: peptone (5 g), malt extract (3 g), NaCl (5 g) and mixed with distilled water 1 L; (v) Medium TSB: casein peptone (17 g), K_2_HPO_4_ (2.5 g), glucose (2.5 g), NaCl (5 g), soya peptone (3 g), and mixed with 1 L ASW; (vi) Medium M1: starch (10 g), yeast extract (4 g), peptone (2 g), and mixed 1 L ASW; (vii) medium MYE: glucose (10 g), yeast extract (3 g), malt (3 g) peptone (5 g), and mixed with 1 L ASW; (viii) medium PYB: peptone (2.5 g), yeast extract (0.5 g), and 1 L ASW.

The best medium for large fermentation was selected based on the result of the antibacterial assay; therefore, MYE was chosen to cultivate the most active strain *Pseudoalteromonas rubra* TKJD 22. The pre-culture was cultivated in a 300 mL Erlenmeyer flask, containing 100 mL MYE medium. After 2 days, the pre-culture was used to inoculate 5 L Erlenmeyer flasks, each containing 1.5 L MYE medium and fermentation was performed in a shaker with 110 rpm at 30 °C for 8 days.

Crude extracts were obtained through separation of cells and supernatant by centrifugation. The cell-free supernatant was extracted with ethyl acetate 1:1 (v/v) and the cell pellet was extracted using MeOH. Both crude extracts were concentrated using a rotary evaporator and tested for antimicrobial activity. The more active cell-free supernatant extract, was used for further purification.

### Isolation and structure elucidation of the active compound

The cell-free supernatant was fractionated using the Interchim Puriflash 4125 Chromatography system applied with a Puriflash C18-HP 30 μm F0080 Flash column. The crude extract was fractionated into 9 fractions using the following gradient: 0–10 min, 20% MeOH; 10–50 min, 20–70% MeOH; 50–60 min, 70–100% MeOH. The activity-guided isolation resulted in 3 fractions active against *E*. *coli* and M. *luteus*, *i*.*e*. F1, F2, and F3, which were further fractionated using Sephadex LH-20 chromatography. The final purification was achieved by HPLC using a semi-preparative C18 column (VP 250/10 Nucleodur C18 Gravity-SB, 5 μm). Analysis of the active crude extract, fractions, and pure compounds was done by HPLC (column EC 250/4.6 Nucleodur C18 Gravity-SB, 5 μm) with the following conditions: gradient 0–10 min, 10% MeOH; 10–40 min, 10%-100% MeOH; 40–50 min, 100%; 50–60 min, 10% MeOH at 1mL/min flowrate. Mass spectra were recorded on a micrOTOF-Q mass spectrometer (Bruker, Billerica, MA, USA) with ESI-source coupled with a HPLC Dionex Ultimate 3000 (Thermo Scientific, Darmstadt, Germany) using an EC10/2 Nucleoshell C18 2.7 μm column (Macherey-Nagel, Düren, Germany). The column temperature was 25 °C. MS data were acquired over a range from 100 to 1000 *m/z* in positive mode. Auto MS/MS fragmentation was achieved with rising collision energy (35–50 keV over a gradient from 500 to 2000 *m/z*) with a frequency of 4 Hz for all of the ions over a threshold of 100. The injection volume was 2 μl with a concentration of 1 mg/mL. The pure compound was analyzed by 1D and 2D NMR measurements in aceton-*d*_6_, as well as by LC-HRMS.

## Results

### Isolation of tunicate-associated bacteria

**Fig 2:**
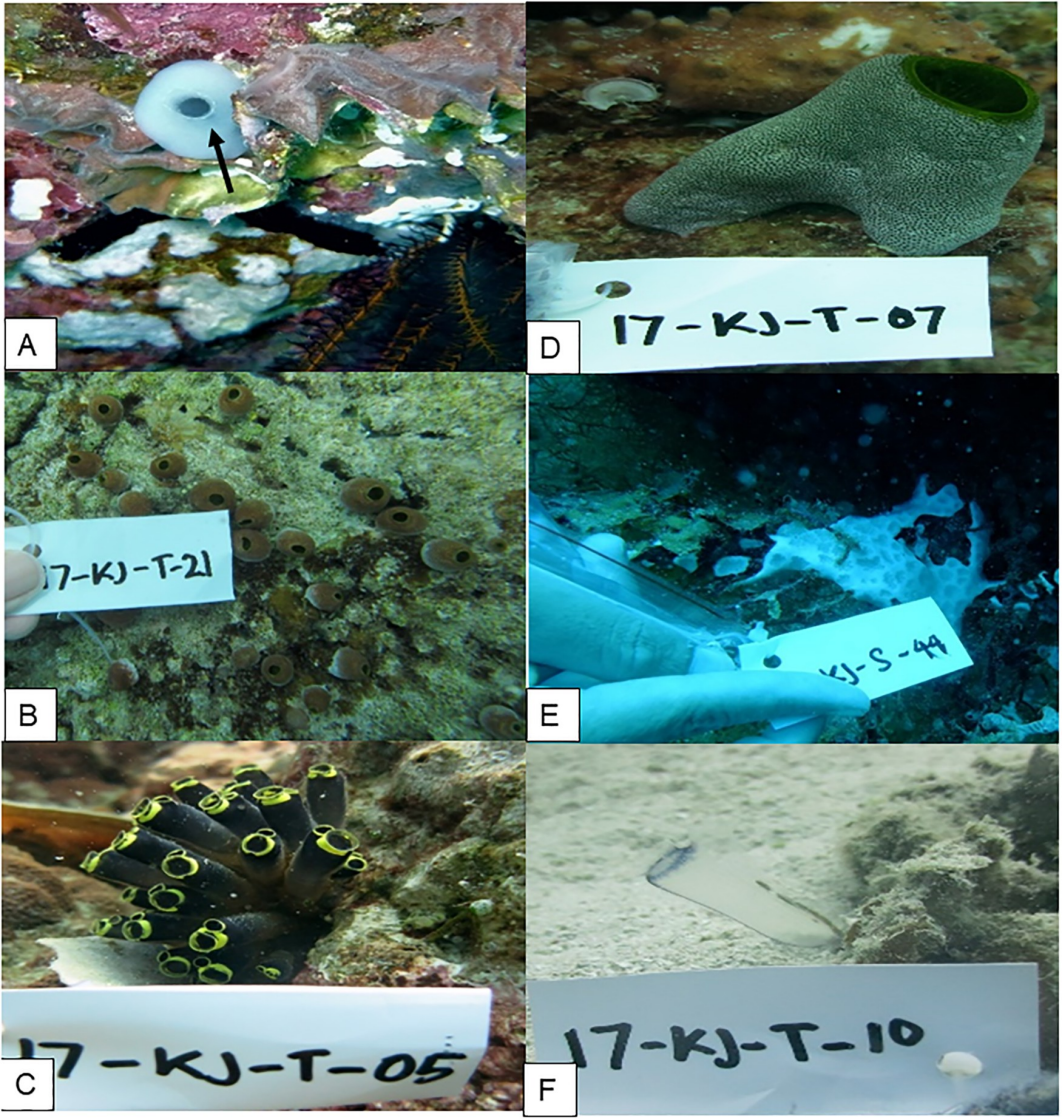
Underwater documentation of the samples. (A) Genus *Ascidia*, (B) genus *Atriolum*, (C) genus *Clavelina*, (D) genus *Didemnum*, (E) genus *Lissoclinum*, (F) genus *Rhopalaea*.

During 11 dives (Fig 1), 37 tunicate specimen were collected (Fig 2). The well-grown and adult zooid was picked from the colony. The preliminary identification of the specimens resulted in the genera *Ascidia* 5.4%, *Atriolum* 13.5%, *Clavelina* 16.2%, *Didemnum* 10.8%, *Lissoclinum* 13.5%, and *Rhopalaea* 18.9%. The rest of the samples (21.6%) remained unidentified (Fig A in S1 File).

Bacteria were isolated from the holobionts, which resulted in 435 axenic strains. From the specimen preliminary identified as *Rhopalaea*, the majority (*i*.*e*., 22%) of the bacterial strains was obtained, followed by *Clavelina*, from which 16% were isolated. The lowest percentage of strains was isolated from *Ascidia* (5%). There was no difference if isolation was performed less than 6 hours from sampling, or if cooled samples were processed after few days in the laboratory, since a share of 52% and 48% of the bacteria was obtained, respectively. In contrast, the media, which have been used for isolation, had an impact on the number of isolated strains. Most bacteria (43%) were isolated on M1 medium (Fig B in S1 File). PYA medium, a medium based on peptone, resulted only in 22% of the strains. Remaining strains were recovered from ISP2 agar plates.

**Fig 3:**
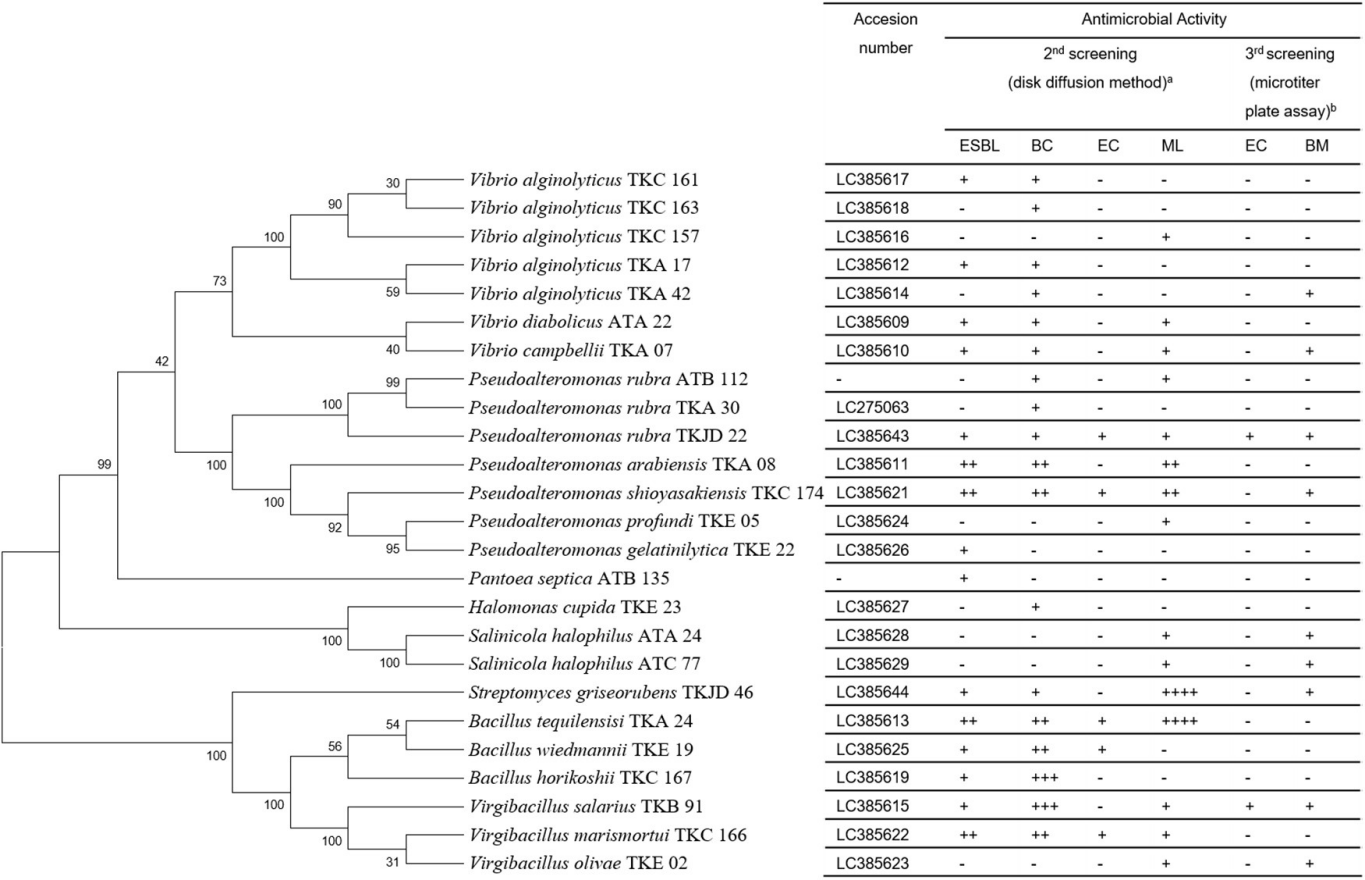
Phylogenetic tree and antimicrobial activities of the 25 active strains. Left side: Phylogenetic tree. Right side: Activity screen with the following test microorganisms: ESBL (Extended Spectrum Beta-Lactamase *Escherichia coli*), BC (*Bacillus cereus*), EC (*Escherichia coli*), ML (*Micrococcus luteus*), BM (*Bacillus megaterium*). a: The signs used indicate:— = no Zone of Inhibition (ZOI), + = ZOI 7–11 mm, ++ = ZOI 12–16 mm, +++ = ZOI 17–21 mm, and ++++ = ZOI ≥ 21mm. b: Signs used indicate:— = no inhibition, + = inhibition (OD_600_ <50% compared to a negative control).

**Fig 4:**
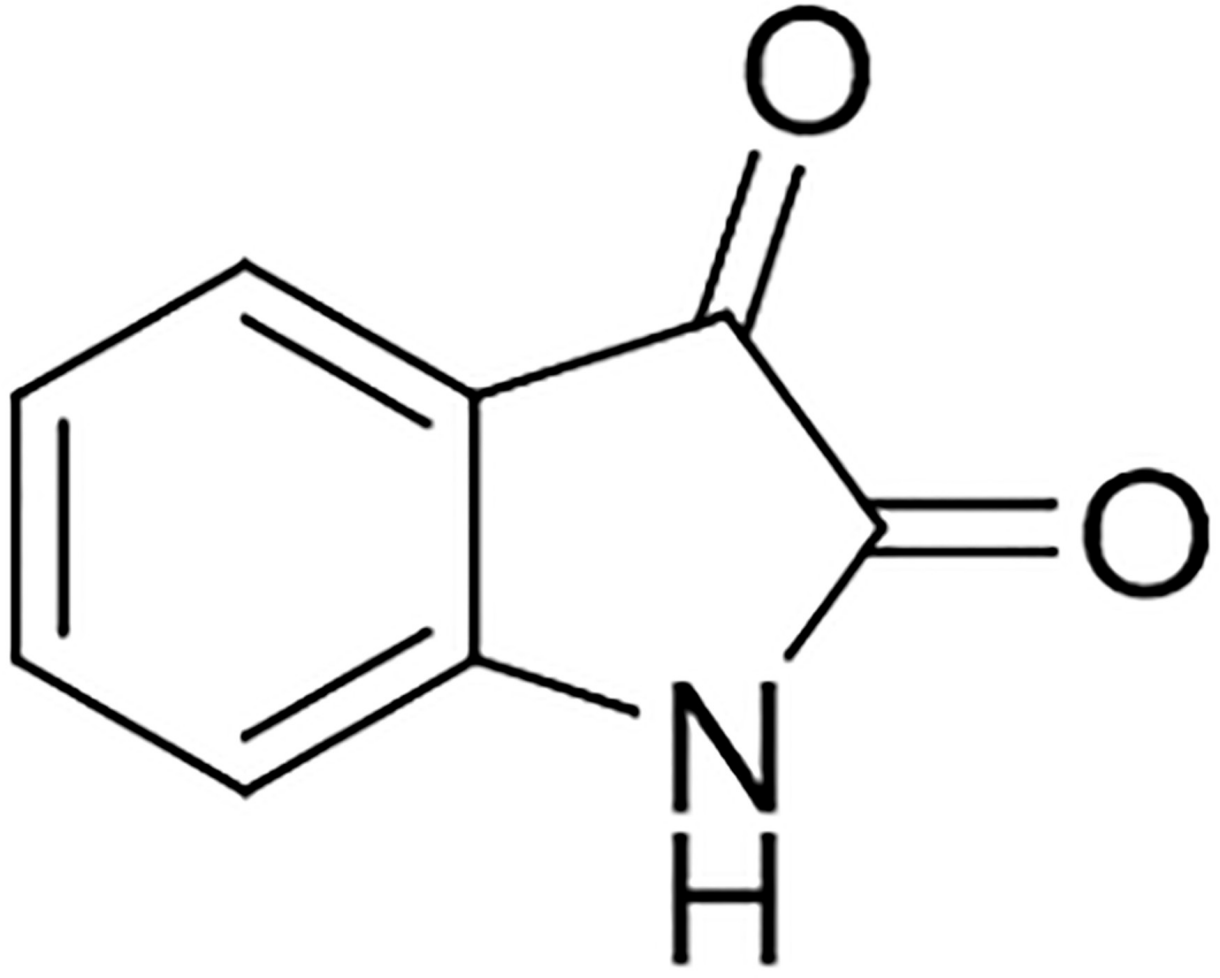
Structure of isatin. Analysis by HPLC and LC-HRMS revealed that fractions F2 and F3 also contained isatin, and therefore these were purified as well. In total 46.8 mg of isatin were isolated from a 9.8 L fermentation. Isatin showed the same moderate inhibitory effect against *B*. *megaterium* DSM32, *E*. *coli* K12 derivative, and ESBL-MDR *E*. *coli* strain, with an MIC value of 200 μg/mL.

After an initial primary activity screening (for details see below; Table A in S1 File), the 16S rDNA gene of the 25 active strains, which showed at least activity against one of the test strains, was sequenced and a phylogenetic tree was generated (Fig 3). Active strains belonged to the following genera: *Bacillus*, *Halomonas*, *Pantoea*, *Pseudoalteromonas*, *Salinicola*, *Streptomyces*, *Vibrio* and *Virgibacillus* (Fig 4). About 2/3 of these strains (n = 16) were Gram-negative bacteria, while the remaining were Gram-positives. In this collection, most of the active strains were classified as *Vibrio* (28%), followed by *Pseudoalteromonas* (20%). *Halomonas*, *Pantoea* and *Streptomyces* were solely represented by one strain (equal to 4%), respectively (Fig B in S1 File).

### Antimicrobial activity

To get a first insight into the antimicrobial activities, all strains were tested against MDR-ESBL *E*. *coli* and MDR *B*. *cereus* using the agar plug method. Thereby, 71 strains (16% of the collection) showed activity (Table A in S1 File) and 45 bacterial strains showed activity against the Gram-negative MDR strain.

The 71 strains, which showed activity in the primary screening, were tested in a secondary screen by evaluating the antibacterial activity of the extracts resulting from liquid fermentation. As a result, 25 out of these 71 strains were positive in the secondary screen and the extract of 13 strains showed activity against both, Gram-positives and Gram-negative MDR-ESBL *E*. *coli* (Fig 3).

The next goal was to identify a compound responsible for the Gram-negative activity observed. Therefore, first, it was tested whether the activity can be linked to a specific peak in HPLC analysis. Hence, 13 active extracts were fractionated using a microtiter plate assay. In this fractionation experiment, only for 9 strains activity was observed. *Pseudoalteromonas rubra* TKJD 22 was prioritized, due to its strong activity against both, Gram-positive and Gram-negative test strains.

### Isolation and structure elucidation of the active compound

The active compound of fraction F1 of *Pseudoalteromonas rubra* TKJD 22 was isolated as red-orange crystalline solid compound. The molecular formula was determined as C_8_H_5_NO_2_ (calculated mass 147.1311 Da) based on the prominent ion peak at *m/z* 148.0410 [M+H]^+^ and *m/z* 170.0240 [M+Na]^+^ in the LC-HRMS (ESI) spectrum. The compound exhibited UV absorption maxima at λ_max_ (MeOH) = 201, 208, 238 and 301 nm (Fig C in S1 File). The structure was elucidated by NMR analysis to be isatin (Fig 4), as well as by comparison with reported literature data [27]. ^1^H NMR (aceton-*d*_6_, 400 MHz) *δ*_H_ 7.62 ppm (1H, td, *J* = 7.77, 1.26), *δ*_H_ 7.53 ppm (1H, d, *J* = 7.49), *δ*_H_ 7.12 ppm (1H, td, *J* = 7.53, 0.85), *δ*_H_ 7.03 ppm (1H, d, *J* = 7.92).

## Discussion

The filter feeding tunicates are exposed to many microorganisms, which enter through the branchial siphon to the atrial siphon [28]. Therefore, it is of utmost importance to the animal that the normal non-pathogenic microbiome is kept intact. It can be speculated that both, the animal and the bacteria, contribute to maintaining and shaping of the microbiome. Bacteria are subject of intra- and interspecies competition. Chemical molecules inhibiting the growth of others in this competition might become lead molecules for drug development based on their antibacterial activities.

Most of the active bacteria isolated from the microbiomes in this project belonged to the phylum proteobacteria, class *γ*-proteobacteria. The highest proportion of *γ*-proteobacteria belonged to the genus *Vibrio* (28%) and *Pseudoalteromonas* (20%). The *Vibrionaceace* family consist of 128 species, with many potential pathogenic species [29]. Many antimicrobial compounds have been reported from this genus, such as andrimid, holomycin, and prodigiosin [29, 30]. Unlike *Vibrio*, which is known for pathogenicity, the genus *Pseudoalteromonas* is well known for production of antibacterial compounds. This genus consists of 41 species, of which 16 are documented as antimicrobial metabolite producers. Around 70 antimicrobial compounds were reported, ranging from alkaloids over polyketides to peptides [31]. The occurrence of the genus *Pantoea* (*Enterobacteriaceae*), which is commonly found in terrestrial habitats, might be explained by cells which were washed into the ocean, *e*.*g*., from the harbor. The strain *Pantoea agglomerans* EH318 was reported as producer of the antibiotic compounds pantocin A and B, which affect mainly other enteric bacteria [32]. Furthermore, broad-spectrum compounds like the antibacterial peptide herbicolin [33], microcin MccEh252 [34], and redox-active phenazines [35] were reported from this genus. In addition, strains of the genera *Halomonas* and *Salinicola* were isolated in this project. However, only few antibacterial metabolites have been reported from the family *Halomonadaceae* to date, *e*.*g*., maminophenoxazinones [36], which renders them interesting for further chemical investigation.

Other active bacteria isolated in this project belonged to the Firmicutes phylum (32%), specifically *Bacillus* and *Virgibacillus* strains. Both genera belong to the family *Bacillaceae*, which showed a high percentage of antimicrobial activity against gram-positive and/or gram-negative bacteria in previous studies [12, 37]. *Bacilli* produce a wide range of natural products, including lipopeptides, polypeptides, macrolactams, fatty acids, polyketides, and isocoumarins, with a series of bioactivities [38, 39]. The genus *Streptomyces* is well known for proliferative producers of specialized metabolites. To date, around 80% of actinobacteria-derived natural products are reported from *Streptomyces* species [40]. Therefore, it was not surprising that strain *Streptomyces griseorubens* TKJD 46 showed activity against MDR bacteria in the screening.

Only 35% (25 of 71) of the active strains cultivated on agar plate retained antimicrobial activity in liquid fermentation, which is in the range generally observed in such bioprospecting projects. However, the fact that only nine strains showed antibacterial activity after fractionation using the 96-well microtiter plate assay, might indicate that some of the activities observed are based on different compounds acting synergistically.

The activity-guided purification of *Pseudoalteromonas rubra* TKJD22 extract resulted in the isolation of isatin, which was first reported in 1841 by Erdman and Laurent as a product from the oxidation of indigo by nitric and chromic acids [41]. In this project, isatin inhibited the growth of *E*. *coli* test strains, a standard lab as well as an ESBL-MDR strain. It was considered to be a synthetic compound for almost 140 years until it was found to be present in plants from the genus *Isatis*, and in secretions from the parotid gland of *Bufo* toads. In humans and other mammals, isatin can be detected as an endogenous molecule. Modified analogues showed stronger activity and good potential against various cancer lines [42]. For example, 6-bromoisatin showed anticancer activity against the human lymphoma cell line U937 with an IC_50_ of 0.02 mg/mL and 5,7-dibromo-*N-*(*P*-methylbenzyl)-isatin showed an IC_50_ of 490 nM against U937 and Jurkat leukemic cell lines [43]. For the compound isatin-3-phenylhydrazone activities against various bacteria and fungi were reported, e.g., *Staphylococcus aureus* (MIC 140 μg.cm^-3^), *Bacillus subtilis* (MIC 45 μg.cm^-3^), *E*. *coli* ATCC95 (MIC 100 μg.cm^-3^), *Pseudomonas aeruginosa* ATCC 2853 (MIC 240 μg.cm^-3^), *P*. *vulgaris* (MIC 200 μg.cm^-3^), and *Candida albicans* (MIC 160 μg.cm^-3^) [44]. Furthermore, the hybrid isatin-indole molecules showed strong antifungal activity, i.e. N’-[(3Z)-1-Benzyl-5-methoxy-2-oxo-1,2-dihydro-3H-indol-3-ylidene]-5-methoxy-1H-indole-2-carbohydrazide against *C*. *albicans* with MIC 3.9 μg/mL, N’-[(3Z)-1-Benzyl-5-bromo-2-oxo-1,2-dihydro-3H-indol-3-ylidene]-5-methoxy-1H-indole-2-carbohydrazide against *Aspergillus niger* with MIC 15.6 μg/mL and N’-[(3Z)-1-Benzyl-5-chloro-2-oxo-1,2-dihydro-3H-indol-3-ylidene]-5-methoxy-1H-indole-2-carbohydrazide against *Penicillium notatum* with MIC 7.8 μg/mL [45]. Majik *et al*. reported the antifouling activity against *Planococcus donghaensis*, *Erythrobacter litoralis*, *Alivibrio salmonicida* and *Vibrio furnisii* of the isatin-based compound (E)-3-(2-Oxopropylidene)indolin-2-one [42].

In general, sessile didemnid species have to protect themselves not only against predators like fish or sea urchin, but also against the settlement by micro- and/or macroorganisms. This can be achieved by chemical molecules like the alkaloids didemnimides A-D, which were reported as chemical weapon of *Didemnum conchyliatum* against predation by carnivorous fish in mangrove habitats [46]. However, the structure of these compounds indicates that they are produced by bacteria. In addition, other molecules produced by cyanobacterial symbionts, *i*.*e*., *Prochloron* and *Synechosystis* species, are beneficial for *Didemnum* [47–49]. It was speculated that cytotoxic constituents produced by these symbionts prevent marine animals to feed on the tunicates [50]. The algal-containing larvae of *Trididemnum solidum* induced vomiting in fish, resulting in a rapid learned aversion to this toxic food source. Furthermore, the patellamides, which are generated by the host-symbiont relationship between *Prochloron* and didemnid ascidian (*Lissoclinum patella*), fulfill this ecological function as feeding deterrents [48]. Therefore, it can be speculated that isatin plays a role in protecting the ascidian against predation due to the cytotoxicity. Hence, the bacteria producing isatin might be beneficial for the tunicate microbiome as defense mechanism.

In this regard, tunicate-associated bacteria can be regarded as promising bioresource for the discovery of biological active compounds, which might have the potential to serve as drug leads for antibiotic development. Further research is required to identify the specialized metabolites used as chemical weapon by the holobiont and to evaluate their potential for development.

## Supporting information

S1 File **Table A. Primary screening for antimicrobial activity from tunicate-associated bacteria against Multidrug resistant (MDR) bacteria. Fig A. Number of collected tunicates sample from each sampling site**. Sampling site 1 resulted 26 samples, sampling site 2 resulted in 7 samples and sampling site 3 resulted in 4 samples. Genus *Rhopalaea* is the highest, followed by *Claveliana* and *Atriolum*. **Fig B. Percentage of tunicate-associated bacteria isolated from different media. Fig C. Percentage of genera with antimicrobial activity**. The genus *Vibrio* is the most active (28%) among others, followed by genus *Pseudoalteromonas* (20%), genera *Bacillus* and *Virgibacillu* sharing the same percentage (16%), and the least number from genera *Halomonas*, *Streptomyces* and *Pantoea* (4%). **Fig D. UV absorption of isatin**.
(DOCX)
