# Supplementary material for: Tunicate-associated bacteria show a great potential for the discovery of antimicrobial compounds
Source: PLoS One. 2019 Mar 15;14(3):e0213797. doi: 10.1371/journal.pone.0213797 (PMC6420000; doi:10.1371/journal.pone.0213797)

**Supporting information**

**Tunicate-associated bacteria show a great potential for the discovery of antimicrobial compounds**

Diah Ayuningrum^1,4,§^, Yang Liu^5,6^, Riyanti^5^, Mada T. Sibero^2^, Rhesi Kristiana^1^, Meezan A. Asagabaldan^1^, Zerlina G. Wuisan^5^, Agus Trianto^2^, Ocky Karna Radjasa^2,3^, Agus Sabdono^2^, Till F. Schäberle^5,6,7,*^

^1^ Department of Coastal Resource Management, Faculty of Fisheries and Marine Science, Diponegoro University, Semarang, Indonesia

^2^ Department of Marine Science, Diponegoro University, Semarang, Indonesia

^3^ Directorate of Research and Community Services, Ministry of Research, Technology and Higher Education, Jakarta, Indonesia

^4^ Tropical Marine Biotechnology Laboratory, Diponegoro University, Semarang, Indonesia

^5^ Institute for Insect Biotechnology, Justus-Liebig-University of Giessen, Giessen, Germany

^6^ Department of Bioresources of the Fraunhofer Institute for Molecular Biology and Applied Ecology, Giessen, Germany

^7^ German Center for Infection Research (DZIF), Partner Site Giessen-Marburg-Langen, Giessen, Germany

^§^ Present address: Department of Aquatic Resource Management, Faculty of Fisheries and Marine Science, Diponegoro University, Semarang, Indonesia

**Table A.** Primary screening for antimicrobial activity from tunicate-associated bacteria against *Multidrug resistant* (MDR) bacteria

| **No.** | **Bacterial Strain** | **ESBL-MDR *E.coli*** | **MDR *B. cereus*** |
| --- | --- | --- | --- |
| 1 | TKA 07 | + | + |
| 2 | TKA 08 | + | + |
| 3 | TKA 09 | + | + |
| 4 | TKA 11 | + | + |
| 5 | TKA 12 | + | - |
| 6 | TKA 17 | + | - |
| 7 | TKA 24 | + | + |
| 8 | TKA 28 | - | + |
| 9 | TKA 30 | + | + |
| 10 | TKA 39 | + | + |
| 11 | TKA 40 | + | + |
| 12 | TKA 41 | + | + |
| 13 | TKA 42 | + | + |
| 14 | TKA 44 | + | + |
| 15 | TKA 52 | - | + |
| 16 | TKA 60 | + | + |
| 17 | TKB 91 | - | + |
| 18 | TKB 101 | - | + |
| 19 | TKB 108 | - | + |
| 20 | TKB 109 | - | + |
| 21 | TKB 111 | - | + |
| 22 | TKB 112 | + | + |
| 23 | TKB 115 | + | + |
| 24 | TKB 123 | + | + |
| 25 | TKC 157 | + | - |
| 26 | TKC 158 | + | - |
| 27 | TKC 161 | + | + |
| 28 | TKC 163 | + | + |
| 29 | TKC 166 | + | + |
| 30 | TKC 167 | + | - |
| 31 | TKC 172 | + | - |
| 32 | TKC 173 | + | - |
| 33 | TKC 174 | + | - |
| 34 | TKC 181 | + | - |
| 35 | TKC 182 | + | - |
| 36 | ATA 22 | + | + |
| 37 | ATA 24 | + | + |
| 38 | ATC77 | + | + |
| 39 | ATB 135 | + | + |
| 40 | ATB 136 | + | + |
| 41 | ATB 170 | + | + |
| 42 | TKD 2 | - | + |
| 43 | TKD 5 | - | + |
| 44 | TKD 10 | - | + |
| 45 | TKD 14 | - | + |
| 46 | TKJD 46 | - | + |
| 47 | TKJD 47 | - | + |
| 48 | TKE 2 | + | - |
| 49 | TKE 3 | - | + |
| 50 | TKE 4 | - | + |
| 51 | TKE 5 | - | + |
| 52 | TKE 6 | - | + |
| 53 | TKE 7 | - | + |
| 54 | TKE 8 | - | + |
| 55 | TKE 10 | + | - |
| 56 | TKE 13 | - | + |
| 57 | TKE 14 | - | + |
| 58 | TKE 15 | - | + |
| 59 | TKE 16 | + | - |
| 60 | TKE 17 | - | + |
| 61 | TKE 19 | + | - |
| 62 | TKE 21 | - | + |
| 63 | TKE 22 | + | - |
| 64 | TKE 23 | + | - |
| 65 | TKE 24 | - | + |
| 66 | TKE 28 | + | - |
| 67 | TKE 33 | + | - |
| 68 | TKE 42 | + | - |
| 69 | TKE 43 | + | - |
| 70 | TKE 44 | + | - |
| 71 | TKJD 22 | - | - |

^1^ESBL = Extended Spectrum Beta Lactamase *Escherichia coli*

^3^BC = Multidrug resistant *Bacillus cereus*

**Figure A.** **Number of collected tunicates sample from each sampling site.**

Sampling site 1 resulted 26 samples, sampling site 2 resulted in 7 samples and sampling site 3 resulted in 4 samples. Genus *Rhopalaea* is the highest, followed by *Claveliana* and *Atriolum.*


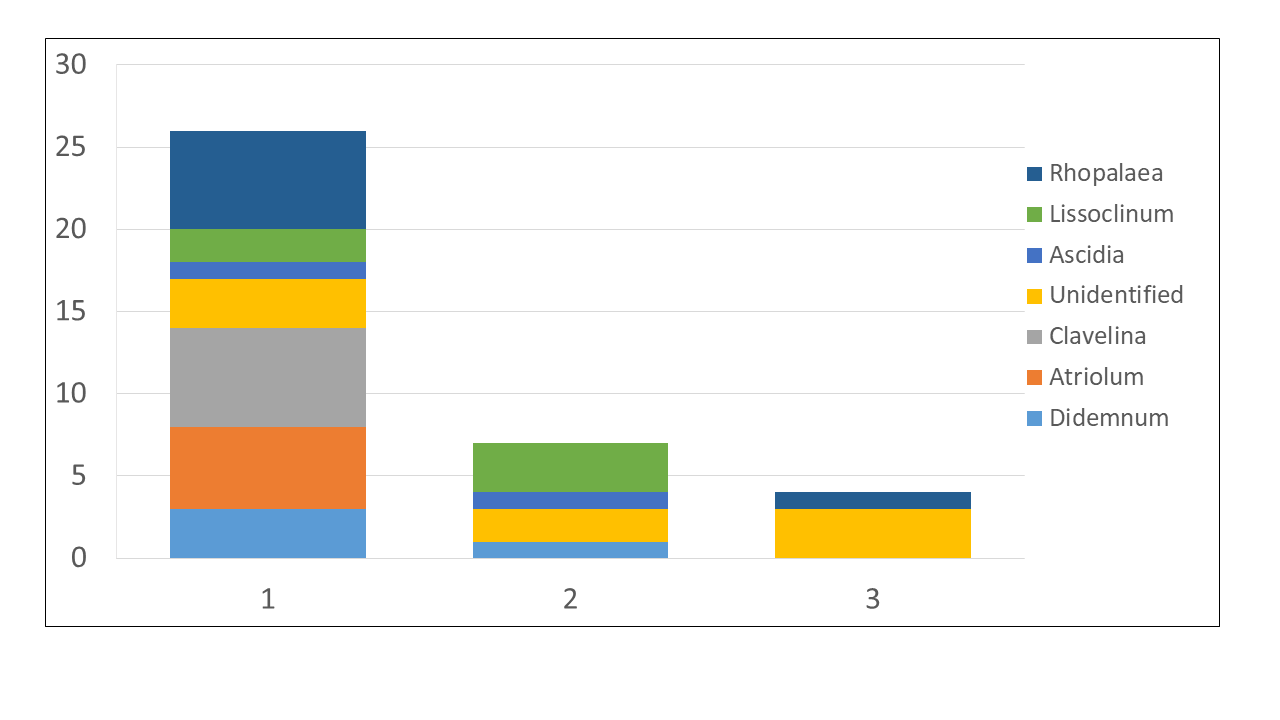


**Figure B.** **Percentage of tunicate-associated bacteria isolated from different media.**

**Figure C.** **Percentage of genera with antimicrobial activity.**

The genus *Vibrio* is the most active (28%) among others, followed by genus *Pseudoalteromonas* (20%), genera *Bacillus* and *Virgibacillu* sharing the same percentage (16%), and the least number from genera *Halomonas, Streptomyces* and *Pantoea* (4 %).

**Figure D.** **UV absorption of isatin.**


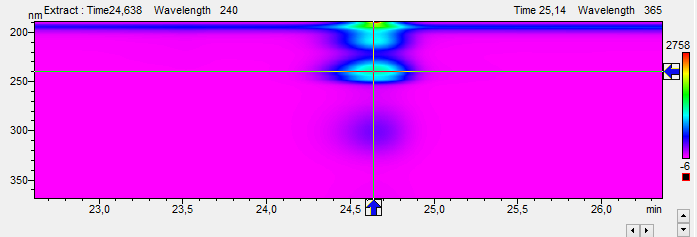

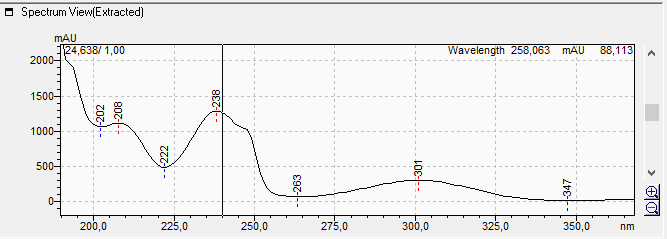

Supplement: S1 File — Table A. Primary screening for antimicrobial activity from tunicate-associated bacteria against Multidrug resistant (MDR) bacteria. Fig A. Number of collected tunicates sample from each sampling site. Sampling site 1 resulted 26 samples, sampling site 2 resulted in 7 samples and sampling site 3 resulted in 4 samples. Genus Rhopalaea is the highest, followed by Claveliana and Atriolum. Fig B. Percentage of tunicate-associated bacteria isolated from different media. Fig C. Percentage of genera with antimicrobial activity. The genus Vibrio is the most active (28%) among others, followed by genus Pseudoalteromonas (20%), genera Bacillus and Virgibacillu sharing the same percentage (16%), and the least number from genera Halomonas, Streptomyces and Pantoea (4%). Fig D. UV absorption of isatin. (DOCX) [file pone.0213797.s001.docx]
